# Editorial: Cognitive and mental health improvement under- and post-COVID-19, volume II

**DOI:** 10.3389/fpsyg.2025.1574083

**Published:** 2025-03-25

**Authors:** Gabriele Nibbio, Yuka Kotozaki, Chong Chen

**Affiliations:** ^1^Department of Clinical and Experimental Sciences, University of Brescia, Brescia, Italy; ^2^Department of Hygiene and Preventive Medicine, School of Medicine, Iwate Medical University, Morioka, Iwate, Japan; ^3^Division of Neuropsychiatry, Department of Neuroscience, Yamaguchi University Graduate School of Medicine, Ube, Yamaguchi, Japan

**Keywords:** COVID-19, anxiety, depression, posttraumatic growth, resilience, family support, students

The COVID-19 pandemic had an enormous impact on a worldwide level: it was officially declared as a global public health emergency by the World Health Organization from January 30, 2020 to May 5, 2023, totaling more than 777 million documented cases and responsible for >7 million deaths (COVID-19 Cases, 2025).

The pandemic also produced a significant impact on mental health (Moreno et al., 2020; Penninx et al., 2022; Duden et al., 2022; Nibbio et al., 2025). Effects on mental health could be related to the biological effects of SARS-CoV-2, particularly as regards cognitive impairment and persistent fatigue (Ceban et al., 2021; Miskowiak et al., 2021; Galderisi et al., 2023; Corbett et al., 2023; Venkataramani and Winkler, 2022; Global Burden of Disease Long COVID Collaborators et al., 2022), but the widespread and pervasive fear of contagion, the increase in social isolation and the severe and prolonged feelings of loneliness and uncertainty also played a critical role in worsening stress, anxiety and depressive symptoms as well as suicidal ideation (COVID-19 Mental Disorders Collaborators, 2021; Chen et al., 2023; Barlati et al., 2021; Salari et al., 2020; Renaud-Charest et al., 2021). Notably, this situation disproportionately impacted individuals in vulnerable and in marginalized groups (Smith et al., 2020; Barlati et al., 2022; Chen et al., 2024) as well as healthcare workers, which often faced severe levels of stress and burnout (Minelli et al., 2022; Fountoulakis et al., 2023; Leo et al., 2021).

Despite the conclusion of the pandemic emergency, the psychological toll and the lingering effects on mental health persist to this day as significant healthcare issues. In this context, gathering and disseminating evidence as well as developing novel insight in the research field represent objectives of relevance in both a scientific and a societal perspective.

The present Research Topic represents the second volume of a collection of works dedicated to cognitive and mental health improvement during and after the pandemic (Chen et al., 2025) and contains nine different manuscripts.

Five studies investigated psychological outcomes in student samples with the use of dedicated surveys.

Gao et al. surveyed 3,049 vocational students in Sichuan Province, China, and reported high rates of poor mental health, anxiety, depression, and insomnia. High family economic status, low stress from the pandemic, and decreased online activity contributed positively to mental health, while the lack of post-pandemic physical activity, disruptions to education and employment, and deteriorating relationships emerged as negative determinants.

Liu et al. surveyed 1,034 college students in Liaoning Province, China, and reported that perceived COVID-19 stress and negative emotions sequentially mediated the negative relationship between perceived social support and sleep quality, while hope and coping styles moderated the sequential mediating effect.

Zeng et al. surveyed 1,555 college students in Hunan Province, China during the first three months of the pandemic. They observed that better family functioning, measured with the Family APGAR Index, was associated with fewer symptoms of depression, neurasthenia, fear, obsessive-anxiety and hypochondriasis.

Wu surveyed 1,711 college students online in Hebei Province, China and reported that social support positively predicted posttraumatic growth during the pandemic and that belief in a just world and meaning in life mediated the relationship.

Jiang investigated 282 secondary vocational school students in Anhui Province, China and reported that self-efficacy was positively associated with resilience and that emotional intelligence partially mediated this relationship.

Two studies relied on interviews of participants to assess psychological outcomes.

Shahwan et al. interviewed 858 adult Singapore residents, reporting that 22% of the sample showed work burnout while 19% showed personal burnout, with younger participants being more frequently burnt-out. Stress was a risk factor, while social support was a protective factor. Path analysis showed that the relation between social support and burnout was partially explained by resilience.

Zhang and Bian interviewed 10 students of Z University, China, and reported that while participants perceived university closed management as a measure enhancing safety and promoting learning engagement, they also emphasized the adverse effects of the pandemic on their physical health, psychology, and social life.

Two studies investigated psychological outcomes in specific populations.

Chen et al. surveyed 327 individuals during the first year of the pandemic in Shanghai, China. 27.8% and 20.5% of participants reported symptoms of depression and anxiety, respectively. Pre-existing health conditions, lack of medical insurance, concerns about shortages of daily necessities during quarantine, and “guilt and self-blame” emerged as risk factors for both depression and anxiety. Moreover, concerns regarding the impact of the epidemic on studies or work and denial were related to depression, while concerns regarding potential rejection or discrimination from the outside world after quarantine were related to anxiety.

Fu et al. conducted a secondary analysis of the longitudinal data of 3,550 adults aged 60 and older who participated in both the 2016 and 2020 waves of the United States Health and Retirement Survey. They conducted a Latent Profile Analysis and a Transition Analysis and found that 42% of the participants reported personality changes during the pandemic. Higher levels of COVID-19 concern were associated with transitioning to Poor-adjusted from Moderate or Well-adjusted categories, while challenges such as healthcare delays and financial hardships hindered transitions from Poor-to Moderate-adjusted and increased the likelihood of Moderate-adjusted individuals transitioning to Poor-adjusted. Finally, Poor-adjusted individuals who provided help to others were more likely to transition to Moderate-adjusted.

The findings from these original studies shed light on the psychological impact of the pandemic across different populations. Several key patterns emerged.

Firstly, the pandemic and related measures, such as lockdowns, have significantly worsened mental health, as evidenced by diverse assessments across various groups.

Secondly, multiple pathways contribute to these negative effects, including concerns about shortages of daily necessities, disruptions in education and employment, financial hardship, healthcare delays, deteriorating relationships, and reduced physical activity. Understanding these pathways can help mitigate the pandemic's psychological toll.

Third, several risk and protective factors have been identified. Demographic factors, such as younger age, and maladaptive coping strategies, including self-blame and denial, are linked to poorer psychological adaptation. In contrast, environmental and psychological factors—such as social support, socioeconomic stability, resilience, hope, a sense of meaning, self-efficacy, and emotional intelligence—serve as protective buffers.

In conclusion, the present Research Topic provides novel insights into both risk elements and protective factors, informing researchers and clinicians on potential targets to contain the impact of adverse effects and, overall, to strengthen psychological resilience.
